# Anti‐microbial peptide gene expression during oral vaccination: analysis of a randomized controlled trial

**DOI:** 10.1111/cei.12848

**Published:** 2016-08-29

**Authors:** M. Simuyandi, M. Kapulu, P. Kelly

**Affiliations:** ^1^ Tropical Gastroenterology and Nutrition GroupUniversity of Zambia School of MedicineLusakaZambia; ^2^ Programme for the Awareness and Elimination of Diarrhoea (PAED)Centre for Infectious Disease Research in Zambia; ^3^ Biological Sciences Department, School of Natural SciencesUniversity of ZambiaLusakaZambia; ^4^ Barts and the London School of MedicineLondonUK

**Keywords:** anti‐microbial peptides, defensins, micronutrients, oral vaccines, zinc

## Abstract

We have observed previously that micronutrient supplementation ameliorated suppression of α‐defensin expression during diarrhoea. However, how interactions between anti‐microbial peptide (AMP) expression and diarrhoeal disease are altered by micronutrient supplementation remain unclear. Using oral vaccination as a model of intestinal infection, we measured changes in AMP expression during multiple micronutrient supplementation. In the first part, volunteers underwent duodenal jejunal biopsy before and at 1, 2, 4 or 7 days after administration of one of three live, attenuated oral vaccines against rotavirus, typhoid and enterotoxigenic *Escherichia coli*. In the second part, participants were randomized to receive a multiple micronutrient supplement or placebo for 6 weeks before undergoing intestinal biopsy, vaccination against typhoid and rebiopsy after 14 days. Expression of human alpha‐defensin (HD)5, HD6, hBD1, hBD2 and LL‐37 was measured by quantitative reverse transcription–polymerase chain reaction. Taken together, the bacterial vaccines, but not rotavirus vaccine, reduced HD5 expression (*P* = 0·02, signed‐rank test) and reduced LL‐37 expression in seven of the eight individuals whose biopsies had expression prevaccination (*P* = 0·03). hBD2 was not detected. In the controlled trial, HD5 and HD6 expression after vaccination was lower [median ratio 0·5, interquartile range (IQR) = 0·07–2·2 and 0·58, IQR = 0·13–2·3, respectively] than before vaccination. There was no significant effect detected of micronutrient supplementation on expression of HD5, HD6, hBD1 or LL‐37. We conclude that live attenuated bacterial vaccines, but not rotavirus vaccine, can reduce intestinal α‐defensins, and typhoid vaccine reduced LL‐37 expression. We found no evidence that micronutrient supplementation in the short term had any impact on anti‐microbial peptide expression.

## Introduction

Diarrhoeal disease remains a major cause of morbidity and mortality in children throughout the tropics and in HIV infection 1, 2, yet host defence against intestinal pathogens remains poorly understood. There is also no clear explanation for the reduced efficacy of oral vaccines in tropical settings. Rotavirus vaccine is half as effective in Malawi as in the United States 3, 4. Oral polio vaccine may have only one‐fifth the efficacy in India that it does in Europe and North America 5. Nutritional impairments have been postulated as possible explanations. Concurrent‐ and/or co‐incident infections may also interfere with induction of responses to vaccine antigens 6.

We have shown previously that human alpha‐defensin (HD)5 and HD6 expression were reduced in adults living in Lusaka, Zambia compared to adults living in London, UK 7. Anti‐microbial peptide (AMP) expression is impaired during human shigellosis 8 and during diarrhoeal disease in Zambian adults 9. Lower expression of HD5 in women appeared to correlate with an increased risk of diarrhoea 10. In animal models, expression of α‐defensins is impaired during salmonellosis 11, yet in mice which are normally susceptible to salmonella protection can be conferred by transgenic expression of a human intestinal α‐defensin 11. Mice which cannot cleave α‐defensins to their active form remain highly susceptible to infection 12. We have demonstrated previously that Paneth cell granule abnormalities are associated with reduced plasma zinc concentrations 13, and we postulated that one possible mechanism of the well‐established beneficial effect of zinc for diarrhoeal disease 14 may be to improve Paneth cell function in terms of increased expression of the α‐defensins HD5 and HD6.

In order to explore further the interaction between micronutrients, intestinal colonization and anti‐microbial peptide‐mediated host defence, we used oral vaccination as a model of intestinal colonization. We describe this work in two stages: exploration of the time–course of changes following administration of three oral vaccines, then a randomized controlled trial of micronutrient supplementation and its effects on these changes. The safety of these vaccines in this trial 15 and the effects of the micronutrient supplement on mucosal architecture have already been published 16.

## Study setting and methods

Two consecutive studies were carried out in adult volunteers in Misisi, Lusaka, Zambia between February 2008 and April 2010. In the first study, three live, attenuated vaccines (Vivotif, ACAM2017 or Rotarix) were given orally to determine the time–course of changes in anti‐microbial peptide gene expression following vaccination. In the second study, participants were randomized to micronutrient supplementation or placebo for 6 weeks prior to administration of one of the three vaccines, Vivotif. In both studies, the primary end‐point was the change in mRNA expression of five key anti‐microbial peptide genes in small intestinal biopsies. Approval for both studies was obtained from the University of Zambia Biomedical Research Ethics Committee (007‐10‐07). The trial was registered as ISRCTN68751738.

### Participants

Volunteers were recruited from a group of 157 residents living in Misisi, Lusaka (the study population has been described previously 13, 15, 17). Inclusion criteria included age between 18 and 60 years and the only exclusion criterion was helminth infection 17. Participants who were pregnant, lactating, had had vaccination within 6 months, had taken antibiotics or non‐steroidal anti‐inflammatory drugs (NSAIDs) within 2 weeks or who had had diarrhoea within 1 month were deferred until their temporary exclusion criterion had elapsed. Informed consent followed a three‐stage process: door‐to‐door notification, focus group discussions and individual counselling leading to written consent.

**Figure 1:**
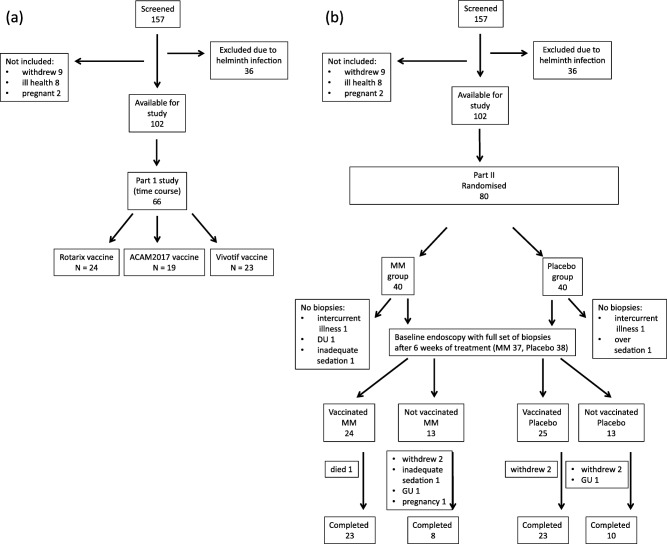
Flow diagrams for the flow of participants through (a) the first study and (b) the second study – the clinical trial. MM = multiple micronutrient; DU = duodenal ulcer; GU = gastric ulcer.

In the first study (Fig. 1a), participants were randomized to receive Vivotif (*n* = 23), ACAM2017 (*n* = 19) or Rotarix vaccine (*n* = 24). Participants underwent enteroscopy under sedation using an Olympus SIF‐10 enteroscope to obtain jejunal biopsies as described previously 10 on the day prior to vaccination and 1, 2, 4 or 7 days afterwards. Each participant underwent endoscopy only twice.

In the second study (Fig. 1b), participants were allocated randomly to receive a multiple micronutrient supplement or a matching placebo for 6 weeks. After this, they underwent duodenal biopsy using a Pentax FG‐29 gastroscope (as the enteroscope was no longer in service). Participants were then immunized with a full course (three doses) of Vivotif typhoid vaccine, and were rebiopsied after 14 days. Randomization, blinding and evaluation of end‐points are explained below. The decision to perform only two endoscopies and collect two sets of biopsies was made to restrict the number of endoscopies to two per participant. In summary, one set of endoscopic biopsies was collected after 6 weeks of trial supplementation, and one after a further 14 days following vaccination, during which period supplementation was continued.

### Vaccines

Vivotif (Ty21a vaccine; Berna Biotech, Bern, Switzerland) is the only licensed oral typhoid vaccine 18, 19. It was administered as a capsule: a single dose was given to those participants scheduled to undergo rebiopsy after a 1‐ or 2‐day interval; two doses were given to those scheduled for rebiopsy after 4 days; and three doses to those scheduled for rebiopsies after 7 days or more. Altogether, 81 participants received Vivotif in parts 1 or part 2. ACAM2017 (Acambis PLC, Cambridge, UK) was derived from a spontaneous labile enterotoxin (LT)‐negative enterotoxigenic *Escherichia coli* (ETEC) isolate from Egypt, which has deletions of the aromatase gene *aroC* and the membrane proteins *ompC* and *ompF*, in addition to its spontaneous deletions of toxin genes for LT, stable enterotoxin (ST) and enteroaggregative ST (EAST). As ACAM2017 has had the gene for CS1 20 added, it expresses the colonization factor antigen II (CFA/II) antigens CS1, CS2 and CS3. In previous studies in London we established that ACAM2017 induces specific mucosal immunoglobulin (Ig)A against CFA/I and CS1, CS2 and CS3 20. The dose of viable organisms in the vaccine vials (3 × 10^10^) was confirmed in the laboratory in three test vials which were then discarded in order to avoid contamination of other vials used for vaccination. The dose contained in each vial was administered as one dose and the vial was then discarded. Rotarix (GlaxoSmithKline, Rixensart, Belgium) is a licensed vaccine against the G1P(8) strain of rotavirus; it was administered as a single dose in 1·3 ml liquid carbonate buffer to all participants, according to the manufacturer's instructions. Allocation to vaccines in the first study was not determined by any clinical criteria, nor was completely random, but was determined by availability at a particular date in the study, previous exposure to vaccines (no participant was given a vaccine which they had received previously) and the temporary exclusion criteria listed above.

### Micronutrient supplementation; randomization and clinical trial design

The composition of the micronutrient (MM) supplement (Immunace; Vitabiotics, London, UK) is shown in Table 1 and compared to recommended nutrient intakes 21. Both MM and placebo were manufactured and packaged in plastic light‐proof bottles labelled only with letters of the four‐letter code (A, B, C, D) held by the manufacturer until the end of the trial, when the databases had been locked. Participants were randomized to one of these four letters using a computer‐generated sequence, so although this four‐letter code was known to the study team, the contents of the bottles and thus treatment allocation was masked from both participants and study team. Participants were given the masked micronutrient or placebo supplement for 6 weeks, then underwent endoscopy for distal duodenal biopsy using a Pentax FG‐29 gastroscope. Following this, they were given Vivotif vaccine (three doses) and rebiopsied after 14 days.

**Table 1:** Micronutrient supplement used

Micronutrient	Daily dose	RNI 21
β‐carotene	6 mg	4·2 mg*
Retinol palmitate	1·6 mg	0·8 mg
Vitamin C	300 mg	40 mg
Vitamin E	80 mg	4 mg
Vitamin D	20 g	10 g^†^
Thiamin (B_1_)	36 mg	0·9 mg
Riboflavin (B_2_)	12 mg	1·3 mg
Pyridoxine (B_6_)	20 mg	1·4 mg
Niacin	54 mg	16 mg
Vitamin B_12_	28 g	1·5 g
Folic acid	1·0 mg	0·2 mg
Vitamin K	140 g	≤ 70 g
Pantothenic acid	40 mg	3 mg
Iron	16 mg	14·8 mg
Zinc	30 mg	9·5 mg
Copper	1 mg	1·2 mg
Selenium	350 g	75 g
Iodine	400 g	140 g
Magnesium	100 mg	300 mg
Manganese	8 mg	1·4 mg
Chromium	200 g	25 g
L‐cystine	80 mg	–
L‐carnitine	60 mg	–
Bioflavonoids	60 mg	–

Composition of Immunace micronutrient supplement produced by Vitabiotics, compared to reference nutrient intake (RNI) for British adults (men or women, whichever is the higher). *Equivalent to 700 μg/day retinol, which is the RNI for adult men. ^†^Based on RNI for older adults, no intake of preformed vitamin D can be quantified as required for adults exposed to sun.

### Analysis of mRNA expression

Intestinal biopsies were collected from jejunum in the first part of the study and distal duodenum in the second study, and processed as described previously 17. Briefly, biopsies were collected into Tri reagent and RNA was extracted. RNA was reverse‐transcribed (RT) using standard techniques and quantified by real‐time polymerase chain reaction (PCR) in a Corbett Rotor Gene 3000 thermal cycler. Five AMP genes were measured: the α‐defensins HD5 (DEFA5) and HD6 (DEFA6), the β‐defensins hBD1 (DEFB1) and hBD2 (DEFB2) and the cathelicidin LL‐37. Cytokeratin‐19 and glyceraldehyde 3‐phosphate dehydrogenase (GAPDH) were used as positive controls for epithelial cells. Supporting information, Table 1 shows sequences of primers used. The RT–PCR conditions are described in Kapulu *et al*. 17; briefly, RT–PCR was performed using SYBR Green (Qiagen, Valencia, CA, USA) over 45 cycles of 95°C, 60°C and 72°C. HD5 and HD6 were expressed as transcripts/μg total RNA using plasmid standards, as described previously 10. cDNA from IL‐1β‐treated Caco‐2 cells was used as a positive control for all the gut RT–PCRs, whereas molecular biology‐grade water was used as negative control. Expression of hBD1, hBD2 and LL‐37 were measured qualitatively (present/absent) in samples in which GAPDH and cytokeratin (CK)‐19 expression were obtained.

### Paneth cell evaluation

Paneth cell counts and morphology were enumerated as described previously 13, 16. Haematoxylin and eosin‐stained sections were prepared of duodenal tissue which had been orientated carefully prior to fixation in the endoscopy unit using a binocular microscope. Paneth cell counts were obtained separately in the crypt base, deep crypt and superficial crypt, and each section was evaluated for the presence of granule depletion which was classified into mild, moderate and severe, as described previously 13.

### Data analysis

Expression of the α‐defensins was quantified absolutely (transcripts/μg total RNA) using a plasmid standard 9, and the change in expression from baseline expressed as ‐fold change (post‐vaccination/prevaccination). Expression of the β‐defensins and LL‐37 were expressed qualitatively (present/absent). The primary end‐point of the first study was the time–course of changes in mRNA expression of the five genes following vaccination. Changes in gene expression between first and second time‐points (‐fold changes) were evaluated using Wilcoxon's signed‐rank test for quantitative expression or by McNemar's test for present/absent expression. The primary end‐point of the second study (the randomized controlled trial) was the change in the mRNA of these genes after vaccination, and comparison of these changes between intervention and placebo groups was made using the Kruskal–Wallis test. In all the graphical representations of data, each individual is represented as one data point for each paired result (before/after vaccination).

## Results

### Effect of vaccination on anti‐microbial peptide expression

**Figure 2:**
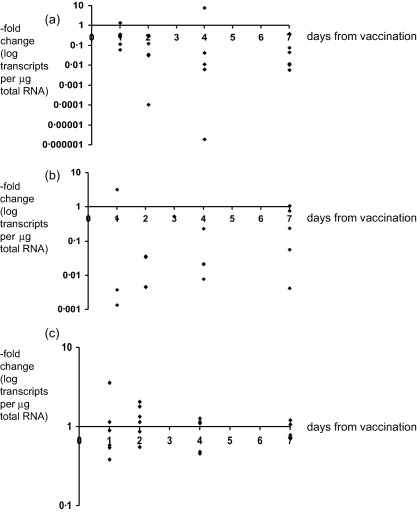
Time–course of changes in HD5 expression following vaccination. Each participant was biopsied twice (prior to vaccination and at one time‐point afterwards) and each point represents the difference in HD5 mRNA between those two biopsies. Note that biopsies collected after 1 day were from participants given only one dose of Vivotif; biopsies collected after 4 days were given two doses; biopsies collected after 7 days were given three doses. There was no significant effect of the number of doses on the response. (a) Time–course of changes in HD5 expression following vaccination against typhoid with Vivotif. (b) Time–course of changes in HD5 expression following vaccination against enterotoxigenic Escherichia coli (ETEC) with ACAM2017. (c) Time–course of changes in HD5 expression following rotavirus vaccination with Rotarix.

In the first study, 64 participants were given one of three vaccines. Their demographic and clinical characteristics did not differ by vaccine allocation (Table 2). However, baseline HD5 expression was higher in the Vivotif group. The time–courses of changes in HD5 for the three vaccines are shown in Fig. 2. There was no significant trend in HD5 and HD6 expression over time following individual vaccines, but administration of either bacterial vaccine (Fig. 2a,b) was followed by a significant decrease of HD5 (*P* = 0·04 by Wilcoxon's signed–rank test). Administration of Vivotif had no effect on hBD1 expression, which remained unchanged in the eight participants who had expression before vaccination, but LL‐37 expression was lost after vaccination in seven of the eight participants who showed expression prior to vaccination (*P* = 0·03 by McNemar's test). Rotarix had no net effect on hBD1 (of 13 who had expression, two lost expression and one gained) or LL‐37 expression (of 22 who showed expression, three lost expression and one gained). ACAM2017 also had no significant effect on hBD1 (one lost and one gained) or LL37 expression (one lost and four gained). hBD2 was not detected in any of the samples. In this part of the work, and throughout both parts, no effect of HIV was demonstrated on expression of these five anti‐microbial genes.

**Table 2:** Baseline data of participants in the first part (time–course)

	Vivotif (*n* = 23)	ACAM2017 (*n* = 19)	Rotarix (*n* = 24)	*P*
Sex (M : F)	8 : 15	10 : 9	10 : 14	0·68
Age (years: median, IQR)	40 (29–50)	42 (35–56)	35 (29–41)	0·09
BCG scar (%)	17 (74)	15 (83)	18 (75)	0·87
HIV seropositive (%)	13 (57)	5 (26)	9 (38)	0·23
CD4 count in HIV positives (cells/μl)	424 (247–845)	307 (243–489)	412 (402–430)	0·31
BMI (kg/m^2^)	22·7 (19·1–26·1)	20·9 (18·7–24·6)	22·3 (19·3–26·9)	0·74
MUAC (cm)	29 (26–34)	27 (26–31)	29 (26–31)	0·68
Fat tissue (% body mass)	32 (26–44)	33 (28–40)	33 (25–38)	0·76
Grip strength (kg)	30·0 (28·0–35·4)	32·1 (26·4–40·4)	28·1 (24·8–34·2)	0·41
HD5 (log transcripts/μg total RNA)	5·3 (4·0–5·8)	4·0 (2·7–4·8)	3·8 (2·9–4·7)	0·002
HD6 (log transcripts/μg total RNA)	4·5 (3·7–5·0)	4·0 (3·6–4·5)	3·8 (2·8–4·4)	0·07
hBD1 (% expression)	8 (35)	8 (42)	13 (54)	0·02
LL‐37 (% expression)	9 (39)	5 (26)	22 (91)	<0·001

*P*‐values refer to the differences in characteristics across all groups. BMI = body mass index; IQR = interquartile range; BCG – bacillus Calmette–Guérin; MUAC = mid–upper arm circumference; HD = human alpha‐defensin; hBD1 = human beta‐defensin 1.

### Effect of micronutrient supplementation on these responses

In the second study (randomized controlled trial), 80 participants were randomized to 6 weeks of micronutrient supplement or placebo administration, which was followed (6 weeks after randomization) by baseline biopsy, then vaccination and then rebiopsy after a further 14 days. Baseline data (obtained after 6 weeks in the trial but before vaccination) showed no difference in clinical characteristics between supplement and placebo groups (Table 3), but HD6 was increased in adults allocated to the control group even before any vaccines were administered. As the first endoscopy was after 6 weeks of supplement or placebo, this baseline difference reflects the effects of the nutritional supplement prior to any vaccine effect. Changes in defensin expression in supplemented and placebo groups after vaccination did not differ by treatment allocation (Table 4).

**Table 3:** Baseline data at date of randomization – clinical trial

	Vaccinees		Controls		*P*
	MM (*n* = 23)	Placebo (*n* = 23)	MM (*n* = 8)	Placebo (*n* = 10)	
Sex (M : F)	8 : 15	8 : 15	4 : 4	1 : 9	1·00
Age (years: median, IQR)	38 (26–41)	42 (34–50)	41 (30–54)	42 (30–48)	0·44
BCG scar (%)	19 (83)	16 (73)	8 (89)	9 (82)	0·74
HIV seropositive (%)	8 (35)	12 (52)	4 (44)	6 (55)	0·79
CD4 count in HIV positives (cells/μl)	424 (247–845)	307 (243–489)	412 (402–430)	313 (245–670)	0·72
BMI (kg/m^2^)	22·3 (20·3–27·4)	22·8 (19·0–25·5)	20·0 (19·9–22·4)	20·3 (19·1–24·3)	0·25
MUAC (cm)	29·5 (27·0–33)	28·0 (26·0–32·0)	27·0 (26·0–32·0)	28·0 (27·0–30·0)	0·71
Fat tissue (% body mass)	34 (29–41)	36 (32–41)	32 (26–38)	31 (28–33)	0·04
Grip strength (kg)	31·3 (27·6–35·4)	32·1 (27·9–35·9)	31·1 (23·6–40·6)	28·1 (23·5–34·1)	0·40

*P*‐value refers to difference between participants allocated to vaccination or not. BMI = body mass index; IQR = interquartile range; BCG – bacillus Calmette–Guérin; MM = micronutrient; MUAC = mid–upper arm circumference.

**Table 4:** Anti‐microbial peptide expression after 6 weeks of trial supplementation

	Vaccinees		Non‐vaccinated		*P* (within vaccinated group)	*P* (within non‐vaccinated group)	*P* (between vaccinated and non‐vaccinated)
	MM (*n* = 23)	Placebo (*n* = 23)	MM (*n* = 8)	Placebo (*n* = 10)			
Before vaccination – baseline values							
HD5 (log transcripts/μg)	6·4 (5·4–6·9)	7·1 (5·9–7·5)	7·2 (6·7–7·4)	7·6 (6·4–8·6)	0·16	0·18	0·03
HD6 (log transcripts/μg)	6·7 (6·1–7·5)	6·9 (6·2–7·4)	6·4 (5·6–7·2)	7·7 (6·7–8·4)	0·87	0·02	0·31
hBD1 (% expression)	7 (30)	8 (35)	2 (22)	2 (18)	1·00	1·00	0·55
hBD2 (expression)	0	0	0	0			–
LL‐37 (% expression)	9 (39)	5 (22)	1 (11)	0	0·34	0·44	0·048
14 days after vaccination – change following vaccination							
HD5 (‐fold change)	0·60 (0·17–4·08)	0·50 (0·07–2·2)	0·12 (0·04–3·8)	0·45 (0·12–1·54)	0·47	0·33	0·36
HD6 (‐fold change)	1·00 (0·22–2·61)	0·60 (0·13–2·29)	1·96 (0·03–5·1)	0·34 (0·22–0·62)	0·36	0·37	0·77
hBD1 (expression gained : expression lost)	1 : 1	0 : 1	0 : 1	0 : 2	1·00	1·00	1·00
LL‐37 (expression gained : expression lost)	2 : 5	4 : 4	2 : 0	2 : 0	1·00	1·00	0·45

Human alpha‐defensin (HD)5 and HD6 expression are shown as median [interquartile range (IQR)] log transcripts/μg total RNA at baseline, and after intervention as median (IQR) –fold change in log transcripts/μg total RNA (up‐regulation > 1·0, down‐regulation < 1·0). MM = micronutrient; hBD1 = human beta‐defensin 1.

### Effect of micronutrients on Paneth cell numbers and granule morphology

Paneth cell numbers and morphology before and after vaccination were evaluated in 19 patients in the placebo group and 15 patients in the micronutrient group. In the remainder, either one of the pair of sections was not suitable for evaluation. Median [interquartile range (IQR)] Paneth cell counts in the basal zone, in biopsies taken prevaccination but after 6 weeks of trial supplements, were 2·2 (1·6, 3·0) in the placebo group and 2·3 (1·5, 3·9) in the micronutrient group (*P* = 0·92). Changes in Paneth cell counts following vaccination were 0·12 (−0·63, 0·58) in the placebo group and 0·13 (−0·46, 0·92) in the micronutrient group (*P* = 0·50). Paneth cell granule depletion was apparent in 12 of the placebo group (two mild, eight moderate, two severe) and 10 (0 mild, six moderate, four severe) of the micronutrient group (*P* = 1·0 by Fisher's exact test).

## Discussion

The ability to augment intestinal innate immunity might have considerable impact upon treatment of persistent diarrhoea which is a common cause, and complication of, malnutrition 22, 23. Given the evidence that Paneth cell function is zinc‐dependent 13, we postulated that a zinc‐containing micronutrient supplement could augment synthesis of α‐defensins, and possibly β‐defensins and LL‐37, in small intestine. This non‐specific effect of vaccines on innate immunity has been shown with oral polio virus and bacillus Calmette–Guérin (BCG) vaccination 24. In view of the difficulties in conducting these studies in children, we carried out this study in adults. We used oral vaccination as a model of colonization, and showed that anti‐microbial peptide expression was down‐regulated after vaccination with bacterial, but not rotavirus, vaccines. We then analysed the effect of micronutrient supplementation in a small Phase II randomized controlled trial, and could detect no effect in preventing the down‐regulation. However, micronutrients increased the expression of HD6 before vaccination in the control group only. It is not clear why this effect was not seen in the vaccination group, as this baseline measurement was made before the vaccines were administered.

We have reported previously that α‐defensin expression is down‐regulated during diarrhoeal disease, and that micronutrient supplementation protected against this 9. A study carried out in Bangladesh at about the same time as our earlier study suggested that hBD2 mRNA (but not peptide) expression is increased during acute diarrhoea (mainly cholera and enterotoxigenic *E. coli*) in adults, and that HD5 peptide (but not mRNA) is reduced 25. Taken together, these data suggest that HD5 expression is reduced by the impact of pathogen colonization. Conversely, the anti‐microbial molecules which are not Paneth cell products appear to be up‐regulated during intestinal infection 25, 26, although we could not detect such an effect in this African population. Further work is needed to resolve this issue.

Based on our earlier study on the effect of micronutrient supplementation on AMP gene expression 9, we hypothesized that the earlier result observed might be demonstrable in a formal randomized controlled trial, and we set up this study to detect an increase in AMP expression over 1–2 weeks. We employed a higher dose of micronutrients than in the previous study, although for a much shorter duration (6 weeks instead of 2 years), but the supplement was given in a trial period leading up to a timed intervention rather than measuring incident episodes of diarrhoea, as in the previous study. However, no effect was seen of the supplementation on α‐defensin mRNA. It is distinctly possible that the duration of supplementation was too short to observe an effect on defensin gene expression, and this remains a limitation of the study. Despite observational data suggesting that Paneth cell degranulation was associated with zinc depletion 13, we were unable to detect an effect of zinc supplementation on Paneth cell granule morphology or on α‐defensin mRNA.

HD5 and HD6 were two orders of magnitude higher in the second part of the study, the controlled trial. We postulate that this was due to using duodenal rather than jejunal biopsies, a change which was necessitated by technical issues with the serviceability of the enteroscope we had used until 2009. The biopsy forceps used with the enteroscope take smaller biopsies from the jejunum than is possible using the larger gastroscopy biopsy forceps for duodenal biopsies. However, comparisons within each part of the study showed consistent effects and we doubt that this reduced the validity of the results.

The micronutrient which we postulated to be likely to have the greatest impact upon innate immunity (Paneth cell characteristics or AMP gene expression) was zinc, but we could detect very little effect. This is consistent with work in piglets, in which zinc had only minimal effects on mucosal innate immunity 27. Other studies have reported effects of vitamin D on LL‐37 expression 28; however, consistent with our previous trial 9, we were unable to detect such an effect. Whether this suggests that baseline vitamin D status is satisfactory in this part of Africa will require further work.

In conclusion, we report here a differential effect on AMP gene peptide expression as a result of vaccine type. These data are consistent with the hypothesis that the nature of the mucosal innate immune response to live attenuated oral vaccines, and the impact of vaccines on anti‐microbial defences, are part of a package of responses which are determined by the nature of the antigens present in the vaccine. Whether such properties can be exploited for rational design of mucosal adjuvants remains to be seen. For instance, rotavirus vaccine confers protection against disease largely through specific IgA secretion into the gut, without a major cell‐mediated immune response 29, 30, while Vivotif induces strong T helper type 1 (Th1)‐dominant cell‐mediated immunity 19. Thus, this might have implications on the efficacy of the vaccines due to the mode of vaccine‐induced protection. We also report that there was no evidence that micronutrient supplementation in the short term had any impact on anti‐microbial peptide gene expression. Perhaps counterintuitively, but entirely consistent with our previous findings 9, 10, HIV had no impact upon anti‐microbial gene expression.

## Disclosure

The authors declare that there are no disclosures.

## Author contributions

P. K. designed the study and obtained grant funding; M. S. and M. K. designed and carried out the experimental work; P. K. wrote the first draft, and all authors revised and approved the final manuscript.

## Supporting information

Additional Supporting information may be found in the online version of this article at the publisher's web‐site:

**Table S1.** Oligonucleotide primer sequences generated used for the detection of mRNA for the genes of interest.
