# Supplementary material for: Anti‐microbial peptide gene expression during oral vaccination: analysis of a randomized controlled trial
Source: Clin Exp Immunol. 2016 Aug 29;186(2):205–13. doi: 10.1111/cei.12848 (PMC5054565; doi:10.1111/cei.12848)
Supplement: Supplementary file 1 — Table S1. Oligonucleotide primer sequences generated used for the detection of mRNA for the genes of interest. [file CEI-186-205-s001.doc]

Supplementary Table 1:

Oligonucleotide primer sequences generated used for the detection of mRNA for the genes of interest.

**Gene of Interest Forward Primer (5’ 3’) Reverse Primer (5’ 3’)**

GAPDH CCAGCCGAGCCACATCGCTC ATGAGCCCCAGCCTTCTCCAT

CK-19 TCGACAACGCCCGTCTG CCACGCTCATGCGCAG

HD5 GCCATCCTTGCTGCCATT GCTTCTGGGTTGTAGCCTCATC

HD6 CCTATGGGACCTGCACTGTCATGG GATGGCAATGTATGGACACACGAC

hBD1 TTGTCTGAGATGGCCTCAGGTGGTAAC ATACTTCAAAAGCAATTTTCCTTTAT

hBD2 CCCTTTCTGAATCCGC GAGGGTCTTGTATCTCCT

LL-37 AGGATTGTGACTTCAAGAAGGAGGACG GTTTATTTATCCAGAGCCCAGAAGC

RORγt GCAGCCAGCTGAGAAGAGTT ATATGGTTCAGGGGCTTGTG

T-bet GATGCGCCAGGAAGTTTCAT GCACAATCATCTGGGTCACATT

GATA-3 GCGGGCTCTATCACAAAATGA GCTCTCCTGGCTGCAGACAGC

FOXP3 GAAACAGCACATTCCCAGAGTTC ATGGCCCAGCGGATGAG

IFNγ ACTGACTTGAATGTCCAACGCA ATCTGACTCCTTTTTCGCTTCC

TGFβ CAGCAACAATTC-CTGGCGATA AAGGCGAAAGCCCTCAATTT

TNFα CCCAGGCAGTCAGATCATCTTC GCTTGAGGGTTTGCTACAACATG

IL-4 ACTTTGAACAGCCTCACAGAG TTGGAGGCAGCAAAGATGTC

IL-5 CCACAAGTGCATTGGTGAAAGA GGAATCCTCAGAGTCTCATTGGC

IL-10 GGCGCTGTCATCGATTTCTT CTCTTGGAGCTTATTAAAGGCATTCT
